# Feasibility of establishing a central repository for the individual participant data from research studies

**DOI:** 10.1186/1745-6215-12-S1-A56

**Published:** 2011-12-13

**Authors:** Catrin Tudur Smith, Kerry Dwan, Mike Clarke, Richard Riley, Douglas Altman, Paula Williamson

**Affiliations:** 1 North West Hub for Trials Methodology Research, Department of Biostatistics, University of Liverpool, UK; 2 All-Ireland Hub for Trials Methodology Research, Queen’s University Belfast, UK; 3 Public Health, Epidemiology and Biostatistics, University of Birmingham, UK; 4 Centre for Statistics in Medicine, University of Oxford, UK

## 

Meta-analysis of individual participant data (IPD) is widely accepted as the most reliable approach for systematic reviews. Advantages include standardising outcome definition across studies, increased potential to investigate subgroups, reducing bias by analysing on an intention to treat basis, minimising the possibility of within study selective reporting, thorough analyses of time to event outcomes, opportunities to identify unpublished studies through collaboration with the original researchers, and incorporating additional follow-up.

IPD provides a rich source of information that allows clinical and methodological developments to extend beyond exploring the main effects that are traditionally of interest in a single trial or systematic review. These opportunities, coupled with the resources required for the IPD approach which are often prohibitive for reviewers, make it essential that as much use as possible is made of IPD that have been collected

We propose that a secure central repository be established to store previously collected IPD. Restricted access to the central repository would only be granted following an approval process that would involve the original reviewers and a nominated committee. The central repository would facilitate exploring additional clinical and methodological questions across a range of studies and reviews.

To assess the feasibility of developing and managing a central repository, we have undertaken an on-line survey of 70 IPD reviewers registered with the Cochrane IPD Meta-analysis Methods Group. We asked about their willingness to provide anonymised IPD from their review and asked about practical issues that this may raise. Non-responders have been reminded about the survey up to three times. Analyses are ongoing and will be presented, along with future plans at the conference.

